# The Effect of Environmental Conditions on the Degradation Behavior of Biomass Pellets

**DOI:** 10.3390/polym12040970

**Published:** 2020-04-21

**Authors:** Hamid Gilvari, Luis Cutz, Urša Tiringer, Arjan Mol, Wiebren de Jong, Dingena L. Schott

**Affiliations:** 1 Section of Transport Engineering and Logistics, Department of Maritime and Transport Technology, Faculty of Mechanical, Maritime and Materials Engineering, Delft University of Technology, 2628 CD Delft, The Netherlands; D.L.Schott@tudelft.nl; 2 Section of Large Scale Energy Storage, Department of Process and Energy, Faculty of Mechanical, Maritime and Materials Engineering, Delft University of Technology, 2628 CB Delft, The Netherlands; Luis.cutz@tudelft.nl (L.C.); Wiebren.deJong@tudelft.nl (W.d.J.); 3 Section of Corrosion Technology and Electrochemistry, Department of Materials Science and Engineering, Faculty of Mechanical, Maritime and Materials Engineering, Delft University of Technology, 2628 CD Delft, The Netherlands; U.Tiringer-1@tudelft.nl (U.T.); J.M.C.Mol@tudelft.nl (A.M.)

**Keywords:** biomass pellets, storage effects, mechanical durability, heating value, equilibrium moisture content

## Abstract

Biomass pellets provide a pivotal opportunity in promising energy transition scenarios as a renewable source of energy. A large share of the current utilization of pellets is facilitated by intensive global trade operations. Considering the long distance between the production site and the end-user locations, pellets may face fluctuating storage conditions, resulting in their physical and chemical degradation. We tested the effect of different storage conditions, from freezing temperatures (−19 °C) to high temperature (40 °C) and humidity conditions (85% relative humidity), on the physicochemical properties of untreated and torrefied biomass pellets. Moreover, the effect of sudden changes in the storage conditions on pellet properties was studied by moving the pellets from the freezing to the high temperature and relative humidity conditions and vice versa. The results show that, although storage at one controlled temperature and RH may degrade the pellets, a change in the temperature and relative humidity results in higher degradation in terms of higher moisture uptake and lower mechanical strength.

## 1. Introduction

Biomass has shown a great potential to meet a significant share of the energy demand in the near future, as one of the main sources of renewable energy [1]. In 2018, up to 10% of the total world energy demand was provided by biomass [2], while it has been estimated that up to 18% of the world’s primary energy demand can be provided only by woody biomass in 2050 [1]. The huge increase in the use of biomass, in particular solid biomass, has raised concerns regarding its transport, storage, and handling, due to its inherent low bulk and energy density and high moisture content [3]. The combination of torrefaction and densification is proved to increase the bulk and energy density and decrease the moisture content of raw biomass [4]. Torrefaction is a thermochemical treatment process, in which biomass is heated at a temperature of 200–300 °C in an oxygen-free environment, and results in the partial decomposition of biomass and removes different types of volatiles, such as carbon dioxide, carbon monoxide, methane, steam, etc. [5,6]. Pelletization is a type of densification process, in which biomass is compressed into cylindrical holes, and it produces pellets with a typical diameter of 3–27 mm and length of 3–31 mm [4]. The global production of biomass pellets has increased more than threefold during the last decade, and reached 55.7 million tons, in 2018 [7]. The main intercontinental trading of pellets takes place between America and Europe. Up to 7.6 million tons of biomass pellets were traded from the USA and Canada to Europe for bioenergy purposes in 2018 [7]. The UK, Denmark, and Italy play a key role in the European biomass pellet import [7].

Large-scale transportation of pellets is mostly performed in bulk. For instance, pellets that are imported from North America to Europe are shipped using large-scale vessels over the Atlantic Ocean [8]. This journey may take a few weeks or a couple of months, depending on the origin, final destination, and terminal time plans [9]. Furthermore, pellets could be stored over a period of weeks before their final use at the end-user storage facilities. In all steps, transport, storage, and handling, pellets are exposed to several mechanical forces (compression, tension, and impact) and drastic changes in temperature and relative humidity (RH), which result in pellet breakage and dust generation, moisture uptake or release, and changes in the calorific value [10,11,12,13].

On the other hand, raw biomass is prone to adsorbing and absorbing moisture from the environment [12], due to the nature of its fibrous structure and presence of hydroxyl groups in the polysaccharides [9,12]. Hereafter, the moisture adsorption and absorption processes will be referred to as moisture uptake. Regained moisture content reduces the mechanical strength of the pellets and affects the heating value [14]. Additionally, pellets with high moisture content tend to produce more fines and dust during transport, storage, and handling activities [12], which in turn increases the risk of self-ignition, results in the loss of a notable portion of bulk, and may cause equipment blockages [3,10]. Moreover, this also creates health problems for people exposed to these conditions [15]. However, the quality parameters of pellets may change, due to variations in environmental conditions. The most relevant quality parameters of biomass pellets in terms of handling, storage, and combustion are the heating value, moisture content, volatile matter, ash content, bulk density, the amount of fines and dust, and mechanical strength [16]. The term “fines and dust” refers to the small particles which are generated either immediately after production or during transport, handling, and storage. The size of the fines and dust may be different in the literature, however, the particle size of smaller than 3.15 mm is a global standard based on ISO standard 17831-1 [17] for determination of the mechanical durability, which is the most common way to determine the mechanical strength of bulk of pellets. According to ISO standard 16559 [18], the mechanical durability is defined as “the ability of densified biofuels units (e.g., briquettes, pellets) to remain intact during loading, unloading, feeding, and transport”. The mechanical durability may be measured using different methods; however, it is usually defined as the mass of fines and dust generated during the experiments to the initial mass of pellets multiplied by 100. The heating value refers to the released energy of the material after combustion. Table 1 presents the effect of storage conditions on some quality parameters of interest of biomass pellets in different storage conditions, as published in the prior literature.

Although it is known from previous studies (Table 1) that uncovered open storage (with direct rain exposure and sun shine) degrades the pellets significantly, [10,11,27], there is not yet a clear guideline for the effect of covered environmental conditions (without a direct rain exposure) on the pellet quality. As shown in Table 1, there are limited sources in the literature addressing the changes in the mechanical durability of biomass pellets using the ISO standard 17831-1 [17] as a global baseline and heating values in various controlled temperature and RH conditions. This paper studies the influence of a wide range of controlled storage conditions (temperature, RH, and storage time) on the equilibrium moisture content (EMC), higher heating values (HHV), and mechanical durability of raw wood and torrefied biomass pellets in bulk. Different storage conditions were designed and executed to mimic various local weather conditions in North America and in the European region, as the main biomass pellet trade happens between these two regions. The main novelty of the present work is to evaluate the effect of sudden changes in the temperature and RH on the pellet properties. This was done by the immediate change in the temperature and RH of the storage conditions from freezing temperature to high temperature and RH and vice versa.

## 2. Materials and Methods

### 2.1. Materials and Measurements

Two types of commercially produced wood pellets and one type of torrefied pellets were studied in this work. The wood pellets were provided from local shops in The Netherlands, where their main application was residential heating. Both types of wood pellets were bought in sealed plastic bags of 10 kg. The sealed bags prevented any moisture uptake to the pellets before starting the experiments. Since the wood pellets were different in color (brown and white), hereafter, we refer to them as brown pellets and white pellets. The brown pellets were made of softwoods residues from the wood industry and certified ENplus A1 [29]. The white pellets were also made of sawdust from the wood industry, but their origin was not disclosed. The torrefied pellets were produced in the UK in a pilot-scale production facility. No information about the densification or torrefaction process for the tested pellets was disclosed.

The proximate analysis of the samples is shown in Table 2. Proximate analysis was determined using a thermogravimetric analyzer (TGA, Thermal Advantage SDT Q600, TA Instruments, New Castle, DE, USA) for determination of fixed carbon, and a muffle furnace (Nabertherm L3/12, Nabertherm GmbH, Lilienthal, Germany) for determination of moisture and ash content. Ash and moisture determinations were performed according to the standards EN 14775 [30] and ISO 18134-2 [31], respectively. Fixed carbon content was determined by the difference between the final residual mass of the TGA experiments and the ash content. Finally, the volatile content was determined by the difference of 100 from the sum of moisture, ash, and fixed carbon. For the TGA runs, 15 mg of samples were placed in an alumina cup in the apparatus and the purge flow rate was set at 50 mL·min^−1^. Experimental runs were performed in an inert nitrogen atmosphere. The TGA runs were executed at a heating rate of 20 °C·min^−1^ up to 900 °C.

The pellet diameter was measured using a digital caliper according to EN standard 16127 [32]. To measure the pellet density, the ends of pellets were sanded to have a uniform surface. Then, the pellet length was measured using a digital caliper. The volume of each pellet was calculated based on diameter and length. The weight of each pellet was measured by using a laboratory balance (PG 1003-S, Mettler Toledo, Columbus, OH, USA, (±0.001 g precision). Finally, the pellet density was calculated by the division of pellet weight to its volume. The pellet density measurement was repeated five times for each pellet type. The bulk density was measured according to the EN standard 15103 [33], using a 5 L cylindrical container.

Before starting the experiments, pellets were kept at laboratory conditions of 20 ± 1 °C and an RH of 60% ± 4%. Temperature and RH were monitored at different time intervals between one day and one week. We characterized the degradation of pellets by the change in the moisture content, HHV, and mechanical durability. 

The moisture content before storage and the EMC after storage for each pellet type at each storage condition was measured according to EN 14774 [34], by placing 300 g of the sample pellets into an oven at 105 °C for 24 h. The EMC ratio was calculated using Equation (1):

(1) $$\mathrm{EMC}\;\mathrm{ratio}=\frac{{EMC}_{\mathrm{AS}}}{{MC}_{\mathrm{AR}}},$$

where $EMC_{\mathrm{AS}}$ is the equilibrium moisture content of pellets after each storage condition and ${MC}_{\mathrm{AR}}$ is the as-received moisture content of pellets. 

The HHV was measured using a bomb calorimeter (Parr 6772, Parr Instrument Company, Moline, IL, USA), using 1 g of the sample pellets following the BS 1016-5 standard [35]. The measurements of moisture content and HHV were repeated twice, and the reported value is the average of the two replications. Table 2 summarizes the physicochemical properties of the pellets studied in the present work before storage, i.e., “as-received”. 

The mechanical durability was measured according to ISO standard 17831-1 [17], using a tumbling can. First, a random sample of materials was sieved with a round hole sieve size of 3.15 mm and 500 ±10 g was weighed and placed into the tumbling can. The device was then rotated at a rotational speed of 50 rpm, for 10 minutes to reach a total of 500 rotations. Finally, the materials were sieved again, using the same sieve to remove the fines and dust from the sample. The mechanical durability was calculated using Equation (2):

(2) $$Mechanical\;durability\;\left(\%\right)=\frac{M_{2}}{M_{1}}\times 100,$$

where $M_{1}$ is the mass of the sieved samples before executing the mechanical durability test and $M_{2}$ is the mass of the sieved samples after the mechanical durability test. The reported mechanical durability results are the mean value of duplicate measurements according to ISO standard 17831-1 [17]. The as-received mechanical durability of the pellets studied in this work is given in Table 2.

### 2.2. Storage Conditions

Pellets were placed in different storage facilities: four climate rooms, one industrial climate chamber, and one home application freezer. A summary of the storage conditions is provided in Table 3. We defined a storage identification code to indicate the temperature and RH in each of the storage facilities. For example, in the storage code “T-19_RH90”, the number next to “T” denotes the temperature (°C) set at the storage facility, while the number after “RH” indicates the relative humidity (%) set for each storage experiment. The conditions in the storage facilities were set to simulate different weather conditions, from freezing temperature to high temperature and high RH. The maximum temperature and RH chosen for this study were 40 °C and 85%, respectively, since higher temperature and humidity values may cause significant off-gassing [36] and physical disintegration to the pellets [19].

Temperature and RH of the climate rooms were controlled every 2 min to ensure a constant temperature and RH. The climate chamber (C+10/600- CTS, Clima Temperatur Systeme, Hechingen, Germany) was used only for 40 °C and 85% RH storage conditions. A freezer (Whirlpool, Benton Charter Township, MI, USA) was used for the storage under freezing conditions. The temperature and RH inside the freezer were monitored once a week using a digital thermometer and an analog humidity gauge, respectively. All the storage conditions were kept constant, except the RH in one of the climate rooms (T5_RH86), where it was arbitrary varied between 72% and 100% (data is shown in Supplementary Figure S1). Therefore, to refer to this storage condition, we use the average RH between the minimum and maximum value, which is 86.

In addition, the effect of storage time was studied for two storage durations, 7 and 30 days, respectively. The maximum storage time was chosen to mimic the duration of travel from the most common pellet exporter ports (e.g., the port of Vancouver) to the EU region (e.g., the port of Rotterdam). Storage time was calculated based on the average speed of bulk carriers and distance between ports. According to Magelli et al. [8], the average speed of bulk carriers is 10 miles.h^−1^. Considering that the distance between the port of Vancouver and the port of Rotterdam is 7170 miles, the whole journey takes around 30 days. On the other hand, 7 days of storage is set to mimic the shorter storage periods, such as storage at the processing plants after production or at the end user’s location.

**Figure 1:**
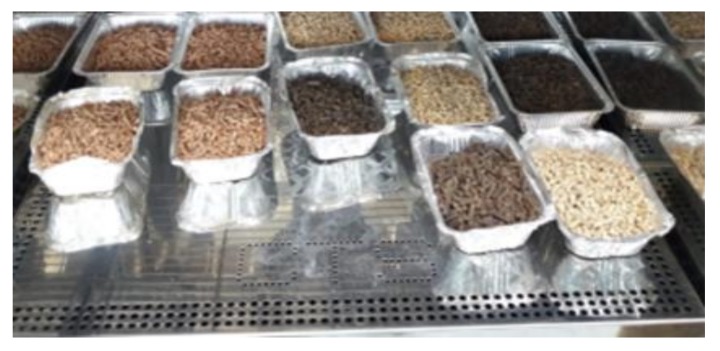
Pellets on aluminum trays in the climate chamber. This figure is an example showing the pellets on aluminum trays. The same trays were used for the other storage conditions.

Inside each storage facility, two batches of 500 g pellets from each pellet type were placed in an aluminum tray without cover (Figure 1). This has been done for each storage time. In total, 76 batches of pellets (38 kg) were stored at different storage facilities.

Two approaches were taken to study the effect of sudden changes in temperature and RH on the properties of the pellets, defrosting and frosting. First, defrosting was studied by storing the pellets in a freezer (T−19_RH90) for 30 days. Then, pellets were transferred (within 30 min) to the climate chamber at 40 °C and 85% RH (T40_RH85) to be stored for another 30 days. Vice versa, for the frosting experiment, we first stored the pellets in the climate chamber at T40_RH85 and then, in the freezer (T−19_RH90). Therefore, the total storage time for either defrosting or frosting conditions was 60 days.

## 3. Results

### 3.1. Moisture Uptake

**Figure 2:**
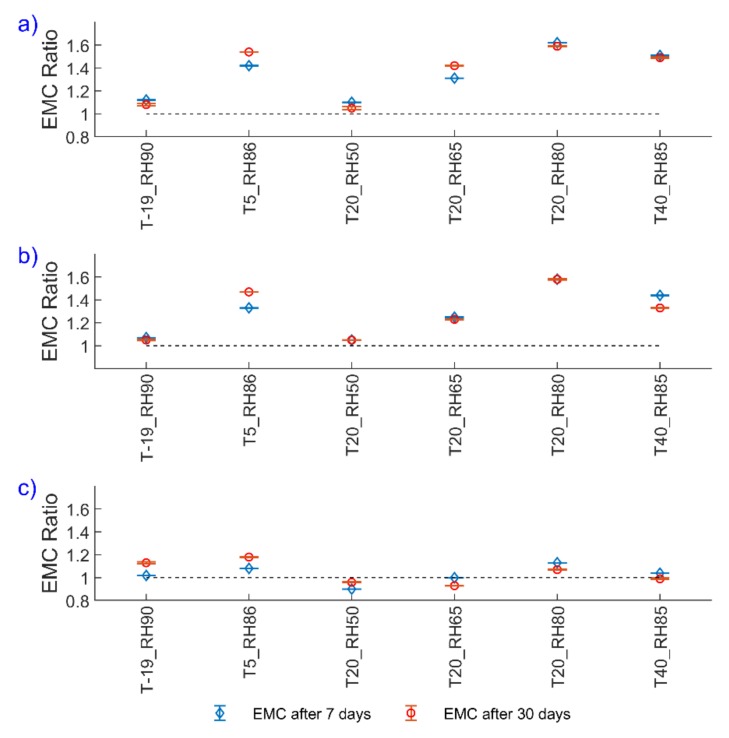
Equilibrium moisture content (EMC) ratio of pellets after 7 and 30 days of storage for (**a**) brown, (**b**) white, and (**c**) torrefied pellets. The error bars show the standard deviation and the dashed lines show the EMC for as-received pellets.

Figure 2 presents the EMC ratio of different types of pellets stored for 7 days and 30 days, at different storage conditions. Results for the EMC ratio indicate that all pellets are already saturated after 7 days, except for T5_RH86 for all pellets, T20_RH65 in the cases of brown pellets, and T40_RH85 in the case of white pellets. In case of T5_RH86, as showed in Section 2.2, the RH varied between 72% and 100%, varying RH seems to be the main reason for non-uniform EMC after 7 days. For the other two cases, the reason has to be further studied, however, the difference in both cases is 0.11% in the EMC ratio.

Lee et al. [14] reported that the EMC is reached after 20 days for wood pellets at temperatures of 25, 35, and 45 °C and Peng et al. [23] reported that the saturated moisture uptake is reached after 10 h for regular and torrefied pellets at a temperature of 30 °C and an RH of 90%. Although it is challenging to compare the saturation time of different types of pellets, due to variations in biomass origins, from the results of this study it can be concluded that the EMC may remain constant at least after 7 days of storage. The EMC results show that torrefied pellets are more hydrophobic than wood pellets. Similar results have been reported before [10,11,12,13,24]. Moreover, we observed a clear relationship between the EMC of wood pellets and RH at the constant temperature of 20 °C. The experimental data at 20 °C were modeled with the Oswin model (Equation (3)), which is shown to be an accurate model for the sorption isotherms of biomass pellets [20].

(3) $$M=a.{\left(\frac{\frac{RH}{100}}{1-\frac{RH}{100}}\right)}^{b},$$

where *M* is the moisture content, *RH* is the relative humidity, and *a* and *b* are the model constants. The results show a high correlation between the EMC and RH of pellets at 20 °C, with R^2^ = 0.900 for brown pellets and R^2^ = 0.997 for white pellets (Supplementary Figure S2). Herein, for wood pellets at T20_RH80 and T40_RH85, we observed that an increase in both temperature and humidity decreases the EMC of brown pellets up to 0.71% and white pellets up to 1.96% with regard to the as-received moisture content. On the contrary, increasing the temperature from 5 to 20 °C results in a slight increase in the EMC of brown and white pellets up to 0.38% and 0.87%, respectively. This suggests a threshold in the temperature for the highest moisture adsorption phenomena (here at 20 °C), however, more data is required to confirm this. Furthermore, when wood pellets are stored at lower temperatures compared to ambient conditions, for example at T-19_RH90 and T20_RH50, the moisture uptake is very low. This can be explained as a combination of low relative humidity (T20_RH50) and decreased movement of water molecules at low temperatures (T-19_RH90) [37]. These findings are consistent with observations made by He et al. [37].

The EMC ratio after defrosting and frosting in the storage conditions was higher, as compared to the EMC in the single storage conditions. EMC ratio increased up to 1.75 for brown and 1.69 for white pellets after defrosting and 1.77 for brown and 1.74 for white pellets after frosting. Considering the stable EMC ratios after 7 days, it is concluded that the higher EMC at defrosting and frosting conditions might be due to a change in the pellet structure, which we noticed by visual observations indicating an increased number of cracks at the surface of pellets, rather than due to the long storage time. Also Graham et al. [11] observed the increased number of surface cracks generated and surface propagation in pellets after six months of outdoor storage.

The fluctuations in the standard deviations of the EMC (Figure 2) suggest that other parameters may also play a role in the results. For example, Whittaker and Shield [38] stated that the main moisture adsorption occurs at the ends of the pellets, because in pelletization process, the outer layer faces the highest heating rate, resulting in the plasticizing and binding of materials to create a polished surface, which in turn preserves the pellet to uptake moisture from the environment. Obviously, the higher the number of particles per batch implies the higher the number of pellet ends. Therefore, the moisture uptake capacity may change due to the number of pellets in a batch. Moreover, existing cracks in the as-received materials may increase the moisture uptake capacity. This requires further study. Although the number of pellets in each batch was not counted in this study, it may explain the fluctuations in EMC results.

### 3.2. Higher Heating Values

**Figure 3:**
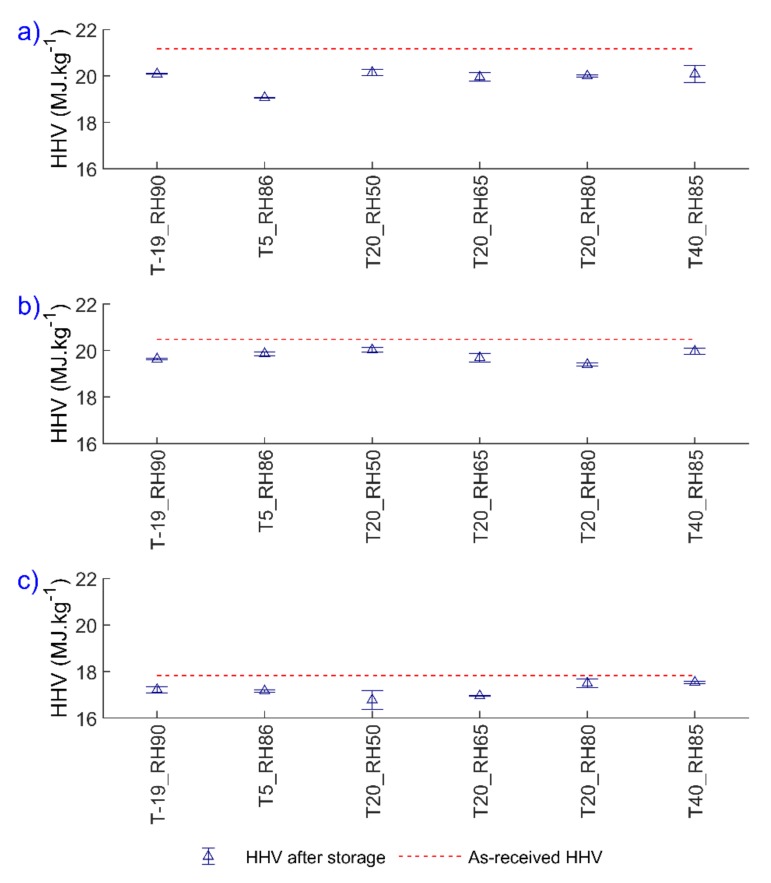
Higher heating values (HHV) of (**a**) brown, (**b**) white, and (**c**) torrefied pellets at different storage conditions after 30 days of storage.

Figure 3 shows the HHV values of three types of pellets after storage at different storage conditions after 30 days of storage. Note that the HHV was not measured after 7 days of storage.

Before storage, the HHV values for brown, white and torrefied pellets were 21.2, 20.5, and 17.8 MJ·kg^−1^, respectively. Note that the HHV of torrefied pellets are lower than the HHV of wood pellets due to the presence of a high amount of ash in the torrefied pellets (Table 2). Results from Figure 3 show that HHV decreased after 30 days of storage, regardless of the storage conditions tested in this study. This may not be only due to the moisture uptake, but also due to potential oxidation of unsaturated fatty acids, as stated by Wang et al. [26]. However, the amount of fatty acids in this work has not been measured. Considering all the storage conditions, the reduction in the HHV for brown pellets was between 5.1% to 10.5% (on average 6.0%), for white pellets between 2.2% and 5.3% (on average 3.5%), and for torrefied pellets between 1.6% and 5.9% (on average 3.5%) after 30 days of storage.

**Figure 4:**
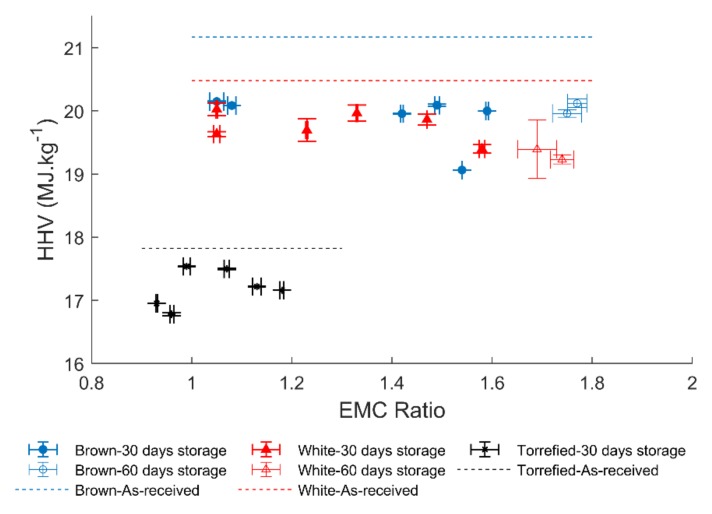
HHV versus EMC of pellets after 30 and 60 days of storage at different storage conditions.

Figure 4 shows the HHV values with respect to the EMC values for all pellets at different storage conditions, including defrosting and frosting conditions. The reduction of the HHV after defrosting was up to 5.7% for brown and 5.3% for white pellets. Meanwhile, for frosting conditions, the HHV decreased up to 4.9% for brown and 6.1% for white pellets. Therefore, defrosting or frosting conditions did not result in a higher reduction of HHV compared to storage at one controlled temperature and RH. In addition, no correlation between the EMC and the HHV was found for all pellets.

### 3.3. Mechanical Durability

**Figure 5:**
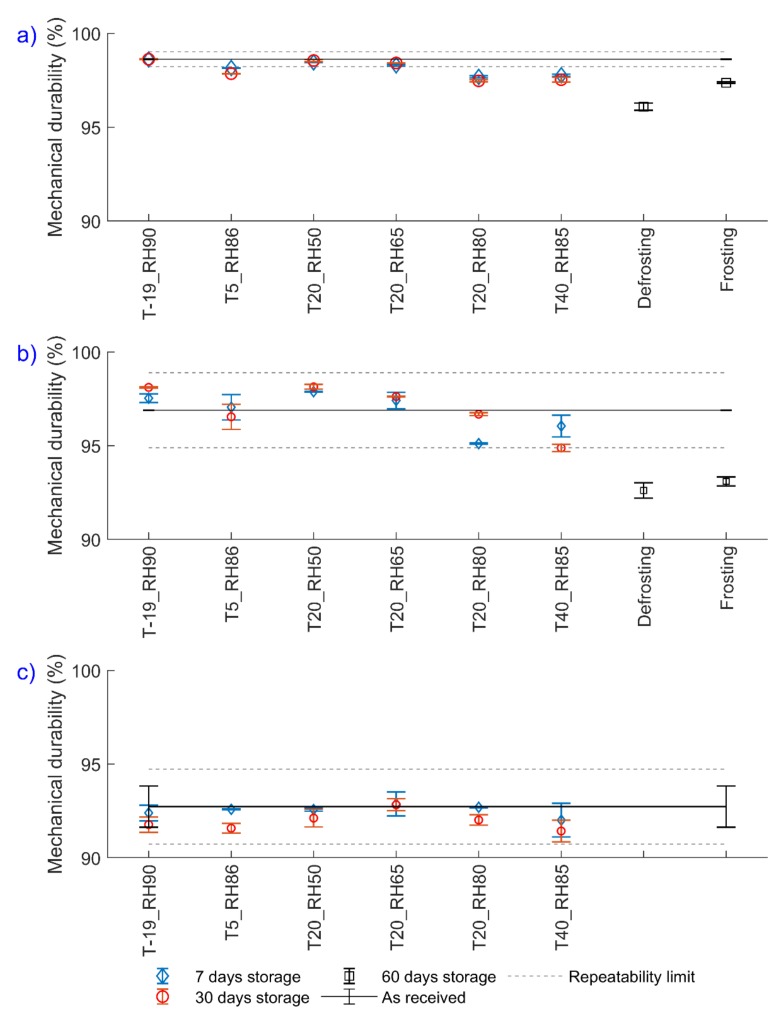
Mechanical durability values of different pellets after storage for (**a**) brown, (**b**) white, and (**c**) torrefied pellets. The error bars show the standard deviations and the solid lines show the as-received mechanical durability.

The results of the mechanical durability tests at different storage conditions are shown in Figure 5. The solid lines show the as-received mechanical durability values, including the error bars at the ends of the lines showing the standard deviations and the dashed-lines show the repeatability limit, which will be defined and explained later in this section. As shown in Figure 5, the as-received mechanical durability for both wood pellets show negligible standard deviations while for torrefied pellets the standard deviation is 1.1%. This might be attributed to the wide heterogeneity in the structure of torrefied pellet which may result in different amounts of fines generated. Even if pellets of the same type are produced under the same conditions, the structure of pellets may significantly differ (Williams et al. [39]). 

For white and brown pellets, mechanical durability was affected mostly when the RH was equal or higher than 80% at a temperature above 20 °C (Figure 5). This occurs due to extended storage and breakage of local bonds in the pellet structure at elevated temperature and RH. By increasing the temperature, water molecule mobility increases [37], so they can diffuse freely within the pellet, causing destruction in the pellet structure. The maximum reduction in mechanical durability was up to 1.2% for brown, 2.0% for white, and 1.3% for torrefied pellets after 30 days of storage.

Defrosting and frosting experiments result in a higher reduction of the mechanical durability of wood pellets. According to the results presented in Figure 5, defrosting the pellets at 40 °C and 85% RH decreased the mechanical durability values up to 2.5% for brown and 4.3% for white pellets. On the other hand, frosting the wood pellets (pellets moved from storage at 40 °C and 85% RH to −19 °C and 90% RH) changes the mechanical durability values up to 1.3% for brown and 3.8% for white pellets. Therefore, defrosting the pellets proves more detrimental for the mechanical durability of wood pellets in comparison with frosting. Moreover, these results can also confirm the results presented in Section 3.1, where the change in pellet structure due to crack generation and propagation at the surface of pellets was observed and reported by visual inspection.

In this study, the mechanical durability was measured using the ISO standard 17831-1 [17]. According to the standard, the repeatability limit is 0.4% for pellets with a mechanical durability value higher than 97.5%, and it is 2.0% for pellets with a mechanical durability value lower than 97.5%. Considering the repeatability limits in the mechanical durability results after storage (Figure 5), it is concluded that for brown pellets (mechanical durability >97.5%) storage at RH higher than 80% results in a significant reduction in mechanical durability value. For white pellets, the mechanical durability changes significantly only if it undergoes defrosting or frosting conditions. For torrefied pellets, although the change in mechanical durability after the storage is 1.3%, this change can be considered insignificant, because all mechanical durability results overlap with the standard deviation of the reference value.

Looking at the changes in mechanical durability, it can be concluded that the pellet quality was either changed or remained constant based on the standard classifications. For instance, the brown pellets which initially met the ENplus A1 certificate may still meet the standard requirement in terms of the mechanical durability. However, as the mechanical durability is not the only standard parameter to be considered for the pellet’s quality, it cannot be concluded whether the pellets keep or meet the standard quality after storage at different conditions. The effect of storage conditions on pellet quality based on the standards requires further research.

## 4. Conclusions

The effect of various storage conditions on the physicochemical properties of two types of untreated wood pellets and one type of torrefied pellets was studied. Results indicate that, regardless of the storage temperature and RH, all the pellets were already saturated after 7 days of storage at constant temperature and RH conditions. Moreover, we found out that the EMC ratio depends on the storage conditions and the type of pellets, since the EMC ratio was obtained between 1.05 and 1.59 for wood pellets and 0.93 and 1.18 for torrefied pellets. Regardless of the storage conditions, the HHV of all the pellets decreased in average by 6.0% for brown pellets and 3.5% for white and torrefied pellets after 30 days of storage at controlled temperature and humidity conditions, which is expected to have great implications in terms of the thermal efficiency and economics of pellet conversion. This highlights the importance of storage conditions for biomass-based pellets. On the other hand, the mechanical durability of pellets was not significantly affected after 30 days of storage, according to ISO standard 17831-1. However, this does not mean that a reduction in mechanical durability is of low importance, because the decrease of mechanical strength, especially at large-scale applications, may have a significant impact on dust and fines generation, which in turn may increase the risk of fire. Furthermore, defrosting and frosting conditions (from freezing temperature to 40 °C and 80% RH and vice versa for 60 days) decrease the mechanical durability of the tested wood pellets up to 4.3% and up to 3.8%, respectively. Moreover, defrosting or frosting conditions resulted in increased EMC and relatively similar HHV, compared to 30 days of storage at constant temperature and relative humidity. To summarize, if possible, a change in the storage conditions should be avoided, in order to keep the change in mechanical durability as low as possible.

## Figures and Tables

**Table 1:** Literature review of the quality parameters of biomass pellets after storage.

Ref.	Type of Pellets	Quality Parameter	Assessment Method	Storage Conditions	Storage Time	Key Results
[10]	Pellets from sawdust, logging residues, and bark	Mechanical durability, Moisture, LHV^2^	6 kg of pellets in an octagonal tumbler, fines were sieved using a 3 mm sieve	20 °C and RH of 85–90%	5 months	-11% increase in moisture uptake -Lower mechanical durability value -No change in the heating values
[11]	White and steam exploded pellets made of softwood and hardwood chips	Mechanical durability	100 g of pellets tumbled in a Dural (II) tester; fines were sieved using a 4.75 mm sieve	Outdoor uncovered or outdoor with covered roof	20 months	-82% drop in the mechanical durability of steam-exploded pellets stored outdoor and 3% drop for white pellets stored indoor
[12]	Untreated and thermally treated birch and spruce pellets	Mechanical durability	ISO standard 17831-1	Outdoor under cover and uncovered	5 months	-High moisture uptake tendency for pellets stored uncovered -Mechanical durability decreased highly in uncovered storage conditions for up to 26% for torrefied pellets and up to 6% for steam explosion pellets -Untreated pellets were totally disintegrated after uncovered storage
[13]	Canola pellets	Mechanical durability	ISO standard 17831-1	Enclosed shed	48 weeks	-Small changes in the mechanical durability
[14]	Wood pellets	LHV^1^	-	15–25 °C	180 days	-Increase in calorific value due to a decreased moisture content
[19]	Softwood pellets	EMC^2^	Weight difference	Up to 93% RH 22°C	10 days	-Linear correlation between the EMC and RH between 15 and 80%
[20]	Spruce, Pine and mixed biomass pellets	EMC	Weight difference	20–90% RH 15 to 25°C	4–8 days	-Temperature has negligible effect on EMC - EMC at high RH depends on pellet type
[21]	Biomass, Cotton stalk, and woody saw mill	EMC	Weight difference	20–80% RH	-	-No difference in EMC of different biomass types at storage up to 70% RH
[22]	Latin species^3^	EMC	Weight difference	40–85% RH	-	-RH and EMC relationships were similar for all biomass samples
[23]	Torrefied wood pellets	EMC	Weight difference	90% RH 30 °C	25 h	-The higher the torrefaction degree, the lower the moisture uptake
[24]	Softwood pellets Torrefied mixed wood Steam exploded pellets	Mechanical durability	100 g of pellets tumbled in a Dural (II) tester, fines were sieved using a 4.75 mm sieve	Various RH and Temperatures	Up to 18 days	-Decreased mechanical durability up to 14% for steam exploded pellets and 70% for white pellets at 90%RH and 30 °C
[25]	8 different biomass pellets	Mechanical durability	ISO standard 17831-1	−28 °C	5 days	-Change in mechanical durability was negligible for pellets with high durability, while for pellets with lower durability, there was a notable decrease in mechanical durability
[26]	Cedar wood pellets	Hardness	Meyer hardness	30–90% RH 30–70 °C	5 days	-Hardness decreased by increasing the RH and temperature
[27]	Wood and torrefied biomass	Dry matter loss	-	95% RH 22 °C	20 days	-White wood are more prone to biological degradation in compare to torrefied pellets
		Mechanical durability, EMC	ISO standard 17831-1	Outdoor	1 year	-Torrefied pellets show less tendency to uptake moisture than wood pellets -Outdoor storage is unsuitable for torrefied pellets
[28]	Pine and recycle wood	Mechanical strength	Three-point bending test	20–95% RH 30 °C	4 days	-Linear relationship between EMC and RH
		EMC	10 g of sample heated at 105 °C for 25 min			-Bulk density and flexural stress decreased with an increased RH
		Bulk density	Using a standard 1 L container			

^1^ Lower heating value, ^2^ Equilibrium moisture content, ^3^ Sorghum stalk, corn stover, wheat straw, and big bluestem.

**Table 2:** As-received physicochemical properties of pellets used in this study.

Pellet Properties	Brown Pellets	White Pellets	Torrefied Pellets
Diameter (mm)	6.1 ± 0.1	6.4 ± 0.1	6.0 ± 0.0
Density (kg·m^−3^)	1209 ± 60	1169 ± 32	1304 ± 40
Bulk Density (kg·m^−3^)	660	600	660
HHV (MJ·kg^−1^)	21.2	20.5	17.8
Mechanical durability (%)	98.6	96.9	92.7
**Proximate Analysis**			
Moisture content (%)	7.2	8	9.3
Ash content (%)	0.7	0.7	16.7
Fixed carbon (%)	17.7	17.9	16.0
Volatile matters (%)	74.4	73.0	58.0

**Table 3:** Summary of the temperature and relative humidity (RH) of the climate chambers.

Storage Code	Storage Type	Temperature (°C)	RH (%)	Example Countries
T−19_RH90	Freezer	−19	90	Sweden, Norway, Finland, Canada
T5_RH86	Climate room	5	86	The Netherlands, Germany, France
T20_RH50	Climate room	20	50	Italy, Portugal, Poland, UK
T20_RH65	Climate room	20	65	
T20_RH80	Climate room	20	80	
T40_RH85	Climate chamber	40	85	Spain, USA, Brazil

## Supplementary Materials

The following are available online at https://www.mdpi.com/2073-4360/12/4/970/s1, Figure S1: RH data at the T5_RH86, Figure S2: Moisture uptake at 20 °C. Experimental results versus Oswin model for (a) brown and (b) white pellets.
