# Supplementary material for: The Effect of Environmental Conditions on the Degradation Behavior of Biomass Pellets
Source: Polymers (Basel). 2020 Apr 21;12(4):970. doi: 10.3390/polym12040970 (PMC7240501; doi:10.3390/polym12040970)
Supplement: Supplementary file 1 [file polymers-12-00970-s001.pdf]

Supplementary materials

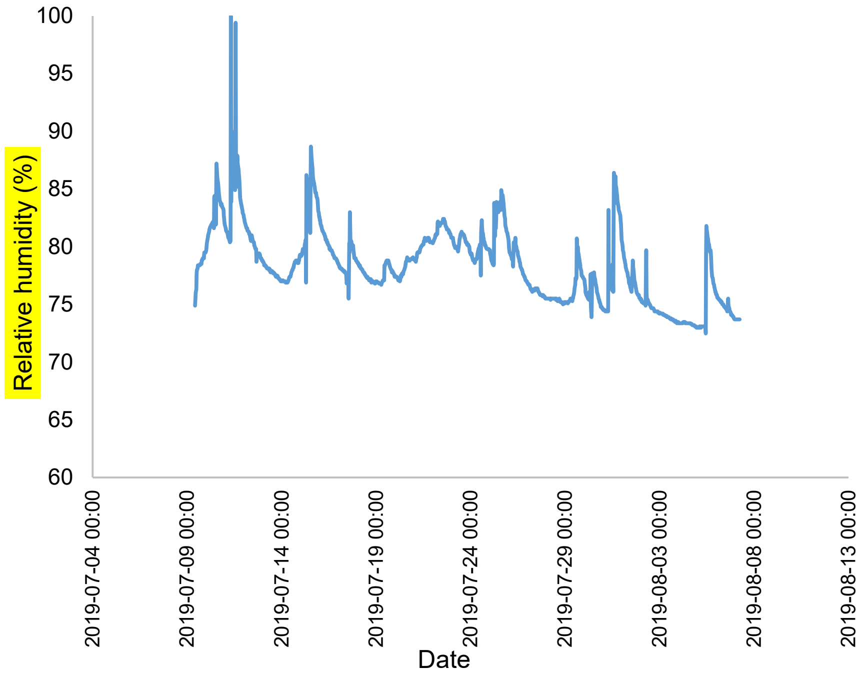

Figure S 1. RH data at the T5_RH86

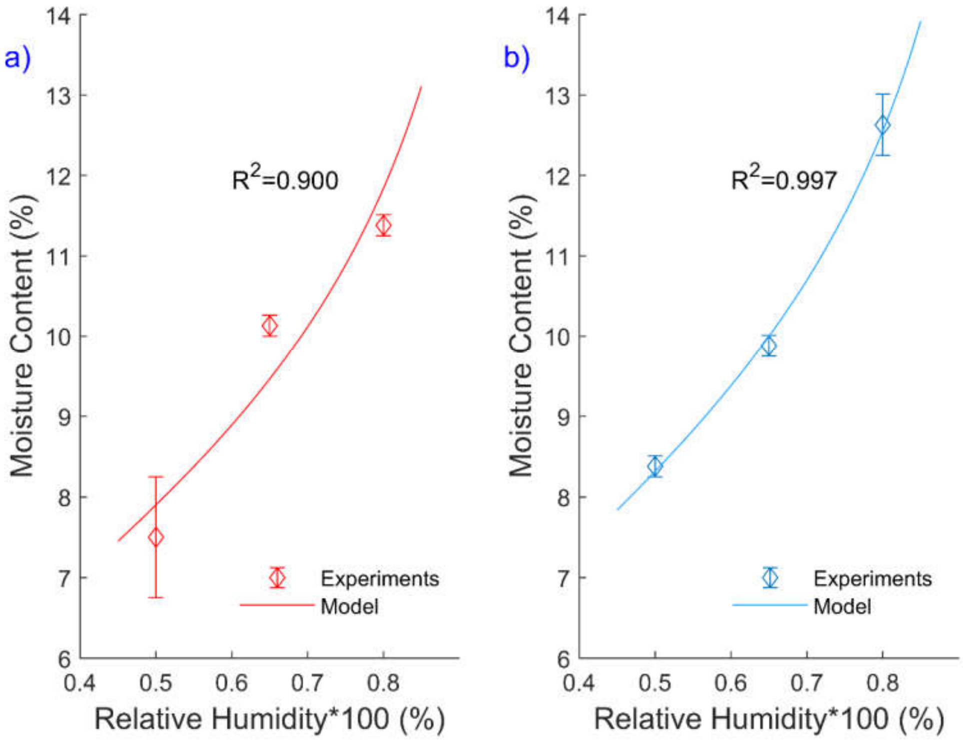

Figure S 2. Moisture uptake at 20°C. Experimental results versus Oswin model for (a) brown and (b) white pellets.
